# Effect of Fructose and Ascorbic Acid on the Performance of Cross-Linked Fish Gelatin Films

**DOI:** 10.3390/polym12030570

**Published:** 2020-03-04

**Authors:** Pedro Guerrero, Iraitz Zugasti, Alaitz Etxabide, Huynh Nguyen Duy Bao, Trung Trang Si, Miriam Peñalba, Koro de la Caba

**Affiliations:** 1 BIOMAT Research Group, University of the Basque Country (UPV/EHU), Escuela de Ingeniería de Gipuzkoa, Plaza de Europa 1, 20018 Donostia-San Sebastián, Spain; iraitz.zugasti@ehu.eus (I.Z.); miriam.penalba@ehu.eus (M.P.); 2 ALITEC, Public University of Navarra, Department of Agronomy, Biotechnology and Food, Campus Arrosadia s/n, 31006 Pamplona-Iruña, Spain; alaitz.etxabide@unavarra.es; 3 Faculty of Food Technology, Nha Trang University, 02 Nguyen Dinh Chieu Street, Nha Trang City 650000, Vietnam; hndbao@ntu.edu.vn (H.N.D.B.); trungts@ntu.edu.vn (T.T.S.)

**Keywords:** fish gelatin, fructose, ascorbic acid, cross-linking, antioxidant

## Abstract

Gelatin was extracted from fish scales in this work, in an attempt to valorise abundant and available fishery by-products as an approach towards a more circular economy. With this strategy in mind, fish scale gelatin was used to prepare active films. In this regard, the development of advanced materials from gelatin involves its modification to enhance functional properties, particularly barrier properties, to achieve the requirements for specific value-added purposes, such as food or pharmaceutical/biomedical applications. The improvement of those functional properties can be achieved by means of chemical cross-linking processes. In this context, non-enzymatic reactions were carried out with the addition of fructose and ascorbic acid into gelatin film forming formulations, and cross-linking was induced by a heat-treatment. These cross-linking reactions resulted in higher barrier features, especially for those films prepared with ascorbic acid.

## 1. Introduction

A substantial part of plastic comes from packaging applications and it is produced from fossil fuels, finite and non-renewable resources. In this scenario, the abundance of bio-waste converts this material into an environmentally friendly option to be exploited as biodegradable and renewable materials [1]. Among them, proteins and polysaccharides have been investigated to develop new biodegradable films due to their abundance, renewable character and film forming ability [2]. In particular, gelatin derived from connective tissues, skin and bones of pig, bovine and fish is getting considerable demand in the last years for applications such as active food packaging and controlled release of bioactive compounds.

Considering that the global fish production peaked at about 171 million tons in 2016 [3], fishery wastes could be an important source of gelatin to develop new materials, reduce food-industry wastes and improve their management, as well as to decrease the use of synthetic plastic products. Currently, the major exporters of fish and fish products, after China, are Norway, Vietnam and Thailand [4]. Particularly, Vietnam produces about 7 million tons of fish per year [5], involving a high quantity of fish wastes, which are rich in collagen and can be used to extract gelatin through acid (type A gelatin) or alkali (type B gelatin) hydrolysis [6]. The amino acid composition of gelatin is similar to that of collagen, containing predominantly proline (Pro) and hydroxyproline (Hyp). In general, the imino acid content (Pro + Hyp) in cold-water fish gelatins is lower than in mammalian gelatins and, thus, they show a lower melting point, which could be a benefit for the manufacture of fish gelatin-based products [7]. 

Gelatin shows some functional properties, such as biocompatibility, film forming ability and biodegradability, that promote its application for food packaging, pharmaceutical and biomedical purposes. However, due to its hydrophilic nature, gelatin-based films exhibit high brittleness and water sensitivity. Therefore, the improvement of those properties must be addressed by means of physical, chemical or enzymatic processes [6]. In this work, non-enzymatic reactions were carried out by adding fructose or ascorbic acid into gelatin film forming formulations and cross-linking was induced by a heat-treatment [8,9,10].

Fructose and ascorbic acid can undergo non-enzymatic reactions, such as Maillard reaction, which involve a series of reactions of the carbonyl group with amino group-containing residues in gelatin, mainly lysine, arginine and histidine [11]. The initial stage of the Maillard reaction with fructose is similar to that with glucose, the carbonyl group of fructose and the ε-amino group of lysine in gelatin react to form a Schiff base, which is isomerized to fructosylamine, resulting in the formation of new species in a process termed as the Heyns rearrangement [12]. Regarding ascorbic acid, in the first stage, 3-deoxy-3-(alkylamino)ascorbic is isolated from the substitution reaction between ascorbic acid and the ε-amino group of lysine. Then, dehydroascorbic acid is obtained from the primary oxidation product of ascorbic acid [13,14]. In a second stage, these products are transformed into reactive intermediates that take part in complex reactions, with a final stage of the Maillard reaction where brown compounds, known as melanoidins [15], are obtained. These final products are anionic chromophoric compounds with some relevant functionalities, including antioxidant, antimicrobial, prebiotic, and antihypertensive activities [10,12,13]. Additionally, ascorbic acid is widely used as an antioxidant compound, providing the final product with an additional functionality [16,17].

In this work, gelatin was extracted from fish scales and it was used to prepare active films with fructose or ascorbic acid in order to promote non-enzymatic crosslinking and improve the functional properties of the resulting fish gelatin films. Hence, the effect of fructose and ascorbic acid on the performance of cross-linked films was assessed.

## 2. Materials and Methods

### 2.1. Materials

Gelatin extracted from fish scales was kindly supplied from Nha Trang University (Vietnam). Glycerol, purchased from Panreac, was used as plasticizer, while D(-)-fructose (VWR Chemicals BDH^®^, West Chester, PA, USA) and ascorbic acid (Sigma-Aldrich, Saint Louis, MO, USA) were used as cross-linkers. 1 N NaOH (Sigma-Aldrich) was used to adjust pH during film preparation. Ethanol 96% (EtOH 96%) from Sigma-Aldrich was used as fatty simulant for the ascorbic acid release assay. Additionally, 1 M picrylsulfonic acid (2,4,6-trinitrobenzenesulfonic acid, TNBS) solution and anhydrous NaHCO_3_ (purity > 99.7%), purchased from Sigma-Aldrich, and diethyl ether stabilized with 6 ppm of BHT and 6 N HCl, supplied by Panreac, were used to measure cross-linking degree.

### 2.2. Extraction of Fish Gelatin

Fresh seabass (*Lates calcarifer*) scales were obtained from Danh Tuyen Co. Ltd. (Nha Trang, Vietnam). The fish scales were packed in polyethylene bags, iced, and quickly transported to the laboratory of Nha Trang University. Then, the fish scales were washed by rinsing with tap water to remove superfluous materials and packed in zip lock plastic bags (500 g/bag). The bags were kept at −20 ± 2 °C until use for gelatin extraction.

Fish scales were immersed and stirred with 5% NaCl solution (1/10, *w/v*) for 30 min at room temperature; this step was repeated twice. The scales were then treated with 10 volumes (*w/v*) of 0.4% NaOH solution at room temperature for 4 h to remove the non-collagen proteins. After the alkali treatment, the scales were neutralized by washing under running tap water. Subsequently, the scales were treated with 10 volumes (*w/v*) of 0.4% HCl solution at room temperature for 4 h to remove the minerals. After acid treatment, the scales were neutralized by washing under running tap water and then subjected to a final wash with distilled water. Gelatin was extracted in distilled water at 70 °C for 1.5 h. The ratio of the scales to distilled water was 1:1 (*w/v*). The coarse solids were filtered out with a filter cloth and the filtrate was then vacuum-filtered with a Whatman No. 1 filter paper. The gelatin solution (vacuum-filtrate) was vacuum-dried in inox trays at 55 °C until dried thin films were formed. The yield of gelatin extracted was 13.2 ± 2.3%.

### 2.3. Characterization of Fish Gelatin

Gel strength was determined using a model CR-500DX Sun Rheometer (Tokyo, Japan) according to the method described in Gelatin Manufacturers Institute of America [18]. Briefly, gelatin was dissolved in distilled water at 60 °C to obtain the final concentration of 6.67% (*w/v*). Gelatin solution was placed in a Bloom jar (capacity of 150–155 mL, overall height of 85 mm, inside diameter of 59 mm, outside diameter of 66 mm, neck inside diameter of 41 mm, shoulder height of 65 mm), and then cooled in a refrigerator at 10.0 ± 0.1 °C for 16–18 h. The gel strength was determined on the rheometer with a cell load of 10 N, test speed of 0.5 mm/s, stroke displacement of 4 mm, and surface detection of 1 g.

Odour of gelatin was determined according to the sensory evaluation method of Muyonga et al. [19] using a five-member panel. Briefly, gelatin was dissolved in distilled water at 50 °C in test tubes with screw caps to obtain the final concentration of 6.67% (*w/v*). The test tubes were held in a water bath at 50 °C, with the screw caps lightly closed. Panelists were trained to assess according to a scale from 0 to 5, in which 0 = odourless; 1 = very mild and only perceivable on careful assessment; 2 = mild but easily perceivable; 3 = strong but not offensive; 4 = strong and offensive; 5 = very strong and very offensive.

Clarity of gelatin solution was determined by the method described in Gelatin Manufacturers Institute of America [18]. Briefly, gelatin was dissolved in distilled water at 65 °C for 10–15 min to obtain a 6.67% gelatin solution in 150 mL beakers. The beakers were held in a 45 °C water bath until sample temperature was 45 ± 1°C. The clarity of a 6.67% gelatin solution was determined at 45 °C by measuring the transmittance percent through a 1 cm cuvette at 620 nm using a model S50 Biochrom Libra spectrophotometer.

Moisture and ash contents of the vacuum-dried gelatin were determined using AOAC official methods number 934.01 and 942.05, respectively [20].

Quantitative and qualitative analysis of free amino acid was performed with a Biochrom 30 + amino acid analyzer physiological system (Bonsai lab, Biochrom, UK).

Fourier transform infrared (FTIR) spectroscopy was carried out by using a Nicolet Nexus FTIR spectrometer equipped with a thermoelectric cell holder and a horizontal attenuated total reflectance (ATR) crystal (ZnSe). The samples were placed directly onto the ATR crystal and spectra were collected in transmittance mode at room temperature. Each spectrum was the result of the average of 32 scans at 4 cm^−1^ resolution. Measurements were recorded in the wavelength range of 4000–800 cm^−1^. All spectra were smoothed using the Savitzky-Golay function. Second-derivative spectra of the amide region were used at peak position guides for the curve fitting procedure, using OriginPro 9.1 software.

### 2.4. Film Preparationc

Firstly, 2.5 g of gelatin and the corresponding amount of fructose or ascorbic acid to reach 10 wt.% and 20 wt.% (on gelatin dry basis) were dissolved in 50 mL of distilled water for 30 min at 80 °C under continuous stirring. After that, pH was adjusted to 7 with 1 N NaOH and then, 10 wt.% glycerol (on gelatin dry basis) was added to the solution, which was maintained at 80 °C for other 30 min under stirring with a final adjustment of pH to 7. Afterwards, 10 g of film forming solution were poured onto Petri dishes (8.6 mm diameter) and left to dry 48 h at room temperature to evaporate water and form the film. After that, films were heated at 70 °C for 24 h and named as 10F and 20F for the films with 10 wt.% and 20 wt.% of fructose, and 10AA and 20AA for the films with 10 wt.% and 20 wt.% of ascorbic acid. Films without fructose and without ascorbic acid, cured at 70 °C, were used as control films. Finally, all films were kept in a controlled bio-chamber (ACS Sunrise 700V) at 25 °C and 50% relative humidity. Film thickness was measured to the nearest 0.080 mm with a handheld QuantuMike digimatic micrometer (Mitutoyo). Three measurements at different positions were taken from seven specimens for each composition. The calculated average thickness was 0.080 ± 0.002 mm.

### 2.5. Assessment of Cross-linking Extension

UV-vis spectroscopy was used to measure light absorption in the UV-vis range (200–800 nm) using a V-630 UV-vis spectrophotometer (Jasco).

The cross-linking extent was measured according to the method of Panzavolta et al. [21]. Briefly, an UV assay of uncross-linked amino groups was performed on thermally treated films and on non-cured films as reference. After the reaction with 0.5% TNBS, gelatin was hydrolysed with HCl (6 M) and extracted with diethyl ether. The solution absorbance was measured against a blank by UV-vis spectroscopy at a wavelength of 346 nm. The moles of free amino groups per gram of gelatin were calculated by the following equation:

$$\left[NH_{2}\right]=\frac{\left(2\times A\times B\right)}{\left(\varepsilon \times b\times x\right)}$$

where A is the sample absorbance, B is the final sample volume (L), Ɛ is the molar absorptivity of TNP-lys, (precisely 1.46 × 10^4^ L·mol^−1^·cm^−1^), b is the cell path length (cm), and *x* is the sample weight (g).

The obtained values of free Ɛ-amino groups/gelatin ratio were employed to calculate the cross-linking extent (CE) equation:

$$CE\left(\%\right)=\left(\frac{{\left[NH_{2}\right]}_{g}-{\left[NH_{2}\right]}_{nf}}{{\left[NH_{2}\right]}_{g}}\right)100$$

where g denotes the non-reacted gelatin used as reference and nf the reacted gelatin.

### 2.6. Characterization of Gelatin Films

Colour parameters (L*, a*, b*) were determined using a CR-400 Minolta Chroma-Meter colourimeter (Konica Minolta). Films were placed on the surface of a white standard plate (calibration plate values: L* = 97.39, a* = 0.03, b* = 1.77) and colour parameters were measured using the CIELAB colour scale: L* = 0 (black) to L* = 100 (white), −a* (greenness) to +a* (redness), and −b* (blueness) to +b* (yellowness). Colour difference (ΔE*) was calculated referred to the non-cured film:

$$\Delta E^{*}=\sqrt{{\left(\Delta L^{*}\right)}^{2}+{\left(\Delta a^{*}\right)}^{2}+{\left(\Delta b^{*}\right)}^{2}}$$

The browning index (BI) was used as an indicator of brown color intensity during cross-linking process and it was calculated as follows:

$$BI(\%)=(\frac{x-0.31}{0.172})100$$

where,

$$x=\frac{\left(a^{*}+1.75L^{*}\right)}{\left(5.645L^{*}+a^{*}-3.012b^{*}\right)}$$

The mechanical properties of fish gelatin films were analyzed using an Insight 10 Electromechanical Testing System (MTS Systems), equipped with a tensile load cell of 250 N. Tensile strength (TS) and elongation at break (EB) were determined according to ASTM D1708-13 [22]. The crosshead speed was set at 1 mm/min and samples with 22.25 mm length and 4.75 mm width were used. Seven samples were tested for each composition.

The ascorbic acid release was determined at room temperature for 4 days by immersion of three samples (1.5 cm × 2.0 cm) of each composition into a EtOH 96% solution (8 mL), used as fatty food simulant. Films were immersed into dark glass vessels to protect the antioxidant compounds from light. UV-vis spectroscopy (V-630 UV-Jasco Spectrophotometer, Jasco, Madrid, Spain) was employed to measure light absorption from 200 to 800 nm. The absorption spectra of the solutions were recorded after 0.5, 1, 2, 24, 48, 72 and 96 h.

### 2.7. Statistical Analysis

Analysis of variance (ANOVA) was used to determine the significance of differences among samples. The analysis was performed with a SPSS computer program (SPSS Statistic 25.0) and Tukey’s test was used for multiple comparisons. Differences were statistically significant at the *P* < 0.05 level.

## 3. Results and Discussion

### 3.1. Characterization of Fish Gelatin

Fish gelatin showed a gel strength of 223.50 ± 0.71 g, an odour value of 1.53 ± 0.21 (odourless = 0; very strong odour = 5), and a clarity value of 95.05 ± 4.26%. Additionally, fish gelatin had a moisture content of 8.37 ± 0.35% and an ash content of 1.06 ± 0.11%. Furthermore, the amino acid composition was determined by elemental analysis and it is shown in Table 1. Glycine (33.15%), alanine (13.58%), proline (11.13%), and hidroxyproline (8.55%) were the most abundant amino acids present in the extracted gelatin, while the lowest amounts were found for tyrosine (0.50%), histidine (0.52%), and isoleucine (0.76%). The relative high content of proline and hydroxiproline is related to the stability of the triple helical structure in renatured gelatin. In particular, hydroxyproline is suggested to play a role as stabilizer of the triple-stranded collagen helix due to its hydrogen bonding ability through its hydroxyl group [23]. The amino acid concentrations determined in this work were similar to the ones obtained by Santos et al. [24].

**Figure 1:**
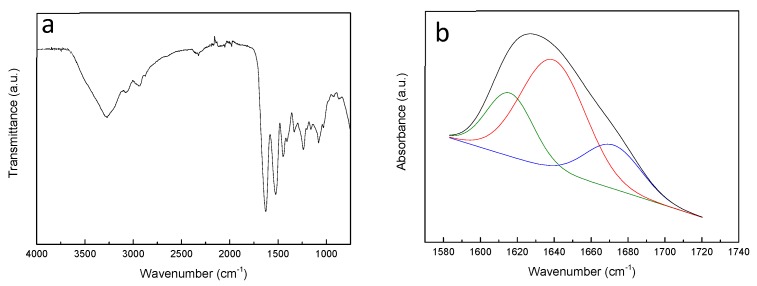
(**a**) FTIR spectra of fish scale gelatin and (**b**) curve fitting spectra of amide I.

The effect of the extraction treatment in protein structure was studied by ATR-FTIR and the spectrum of extracted gelatin is shown in Figure 1a. As can be observed, the main absorption bands of gelatin were related to C=O stretching at 1630 cm^−1^ (amide I), N–H stretching at 1530 cm^−1^ (amide II) and C–N stretching at 1230 cm^−1^ (amide III) [8,25].

Gelatin is a semi-crystalline biopolymer with both crystalline and amorphous phases. The crystalline phase consists of triple helixes and bundles of triple helixes of denatured collagen, whereas the amorphous phase is composed of colloidal chains which form simple helixes due to the high content of proline, responsible for protein chain turns [26,27,28]. Therefore, in order to assess the secondary structure of the extracted fish gelatin, curve fitting of amide I band was carried out (Figure 1b). The absorbances at 1615.92, 1642.36, and 1675.06 cm^−1^ are assigned to β-sheet, α-helix/random coil, and β-turn conformations, respectively [8]. From the analysis of Figure 1b, it was found that the secondary structure of gelatin consisted mainly of α-helix/random coil conformation (56.87%), while β-sheet (23.44%) and β-turn (19.70%) conformations were present to a lesser extent.

### 3.2. Assessment of Cross-linking Extension

It is generally agreed that the rate of non-enzymatic reactions increases at temperatures over 55 °C and, therefore, most of the studies dealing with these reactions are carried at temperatures higher than 55 °C, with a significant enhancement of the cross-linking extension as heating temperature increases [10,29,30]. Taking this into account, a preliminary characterization was carried out by UV-vis spectroscopy (data not shown) in order to establish an appropriate heating temperature to promote the cross-linking reaction. In this study, the temperature selected was 70 °C.

**Figure 2:**
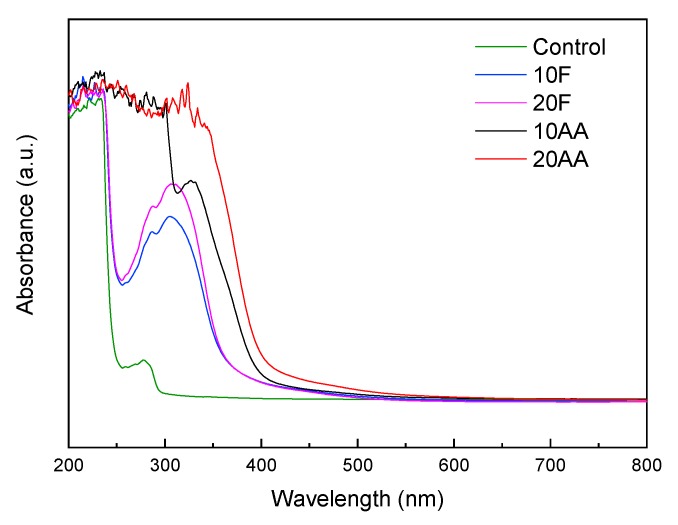
UV-vis spectra of fish gelatin films as a function of cross-linker type and content.

UV-vis spectroscopy was used to study the reaction extension as a function of the type of cross-linker (fructose or ascarbic acid) and their content (10 and 20 wt.%). As shown in Figure 2, fish gelatin films (control) reveled high absorption of UV light due to the presence of peptide bonds (200–250 nm) and aromatic amino acids (250–300 nm), such as tyrosine and phenylalanine [31]. Regarding the cross-linker addition and subsquent heat-treatment, an absorbance increase was shown, irrespective of the type of cross-linker. As the increase in UV light absorbance is often used as a measurement of cross-linking extension, the high absorption at 300–420 nm would be indicative of the reaction between gelatin and the cross-linkers used in this work [32]. Taking this into account, it can be said that fructose-cross-linked films showed lower cross-linking extension than gelatin films cross-linked with ascorbic acid, since lower absorbance values were found for fructose-added films. Furthermore, these results indicated that higher cross-linker amount induced a more extensive cross-linking reaction. It is worth noting that all cross-linked films presented excellent UV light barrier properties, which could prevent undesirable oxidation reactions, for instance, in food products.

In order to support the previous results, TNBS method was used to determine quantitatively the degree of cross-linking in the films containing different contents of fructose or ascorbic acid and values are summarized in Table 2. Results indicated that gelatin was glycated by fructose and ascorbic acid, as previously shown by UV spectroscopy. As can be seen in Table 2, a higher cross-linker content led to a higher reaction extension for both cross-linkers. Although the reaction extension was not significantly (*P* > 0.05) different for the films cross-linked with 10 wt.% fructose or ascorbic acid, the highest cross-linking extension was found for the films cross-linked with 20 wt.% ascorbic acid, in accordance with UV-vis spectra shown in Figure 2.

### 3.3. Characterization of Gelatin Films

**Figure 3:**
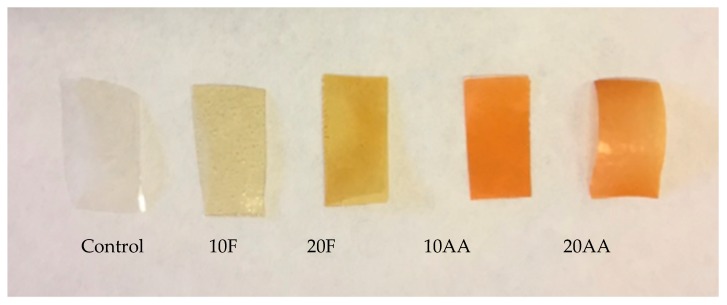
Colour change of fish gelatin films as a function of cross-linker type and content.

The colour change that takes place in the cross-linked films after heating can be considered as an indicator of the cross-linking reaction. Therefore, the reaction extension between gelatin and fructose or ascorbic acid was evaluated according to the colour change that took place in the films after the heating treatment (Figure 3).

Additionally, colour parameters were measured according to CIELab scale and values are shown in Table 3. The addition of cross-linkers caused a decrease in L* and a* values, while b* value increased and, consequently, total colour difference (ΔE*) significantly (*P* < 0.05) increased in all samples but, especially, in fish gelatin films with 20 wt.% ascorbic acid, indicating that ascorbic acid induced a higher extension of the reaction than fructose, as shown by CE values in Table 2.

Due to the formation of brown-coloured products in the final stage of cross-linking reactions, the browning index (BI) was calculated to study the extension of the reactions. As can be seen in Table 3, both cross-linkers induced browning, irrespective of type and content. However, ascorbic acid-cross-linked films presented significantly (*P* < 0.05) higher values of BI, especially those films prepared with 20% of ascorbic acid, suggesting that a higher amount of brown-coloured products was formed and so, indicating that ascorbic acid promoted a more extended cross-linking than fructose.

Once the cross-linking extension was indirectly and directly analyzed as a function of cross-linker type and content, mechanical properties were measured in order to assess the effect of cross-linking on the film performance. Hence, mechanical behaviour was evaluated, and tensile strength (TS) and elongation at break (EB) values of fish gelatin films are shown in Table 4. It is well known that the mechanical properties depend on the interactions between the components [33,34,35]. Therefore, TS values significantly (*P* < 0.05) increased with the incorporation of 10 wt.% fructose or ascorbic acid due to the cross-linking reaction; however, a TS decrease was observed from 10 to 20 wt.% of cross-linker, probably due to a higher content of water formed during cross-linking reaction. Since water acts as a plasticizer, TS decreased but EB significantly (*P* < 0.05) increased, especially for those gelatin films cross-linked with 20 wt.% of ascorbic acid, the films that showed the highest cross-linking extension.

**Figure 4:**
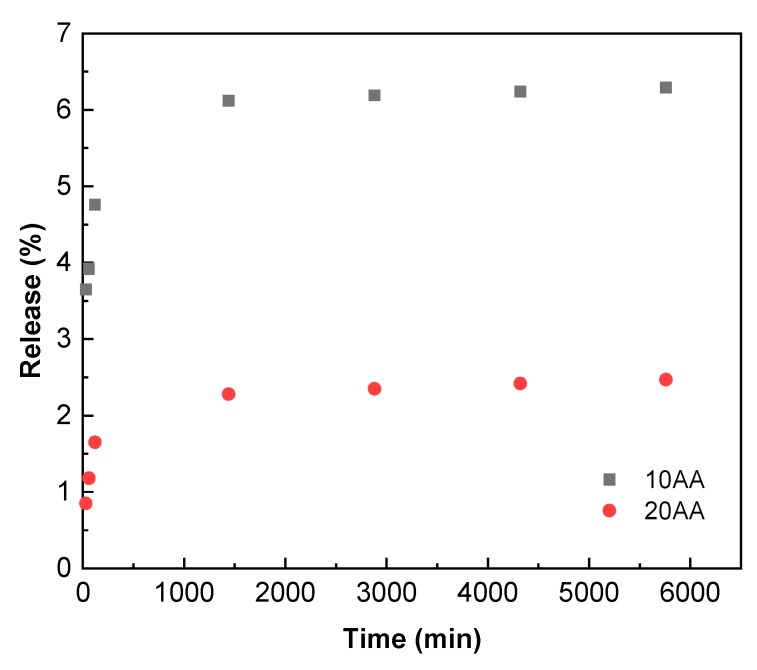
Release of ascorbic acid from gelatin films as a function of acid content and immersion time.

Finally, the release of ascorbic acid from fish gelatin films was measured. As can be seen in Figure 4, the release was higher for the films with 10 wt.% of ascorbic acid, in accordance with a lower cross-linking extension and, thus, a higher amount of free ascorbic acid that can migrate from the film into the fatty food simulant. Additionally, since a higher cross-linking extent is usually related to a more compact structure [36], this hinders the antioxidant release, as observed for the films with 20 wt.% of ascorbic acid.

## 4. Conclusions

Fish gelatin films prepared with 10 and 20 wt.% of fructose or ascorbic acid were homogeneous and transparent. After a heating treatment at 70 °C, a Maillard reaction was promoted and cross-linking extension was assessed by UV-vis spectroscopy, which showed an intense absorbance above 300 nm related to the formation of brown compounds during cross-linking. In this regard, the films with 20 wt.% ascorbic acid showed the highest cross-linking extension, as confirmed by the highest ΔE* and BI values. Additionally, the formation of water involved in the cross-linking process caused an increase of the film flexibility due to the role of water as plasticizer. Finally, the ascorbic acid release from the films into a fatty simulant was analysed and it was observed that the consumption of the acid in the crosslinking reaction caused a limited antioxidant release.

## Figures and Tables

**Table 1:** Amino acid content of fish gelatin expressed as number of residues per 100 residues.

Amino Acids	Concentration (%)
Hydroxyproline	8.55
Aspartic acid	4.28
Threonine	2.19
Serine	2.85
Glutamic acid	7.16
Proline	11.13
Glycine	33.15
Alanine	13.58
Valine	1.77
Methionine	1.36
Isoleucine	0.76
Leucine	1.73
Tyrosine	0.50
Phenylalanine	1.52
Histidine	0.52
Lysine	2.62
Arginine	4.96

**Table 2:** Cross-linking extension (CE) of gelatin films as a function of cross-linker type and content.

Film	CE (%)
10F	37.58 ± 0.48^a^
20F	59.72 ± 1.12^b^
10AA	37.88 ± 1.32^a^
20AA	65.62 ± 1.15^c^

^a–c^ Two means followed by the same letter in the same column are not significantly (*P* > 0.05) different through the Tukey’s multiple range test.

**Table 3:** CIELab parameters (L*, a*, b*), total colour difference (ΔE*) and brown index (BI) for gelatin films as a function of cross-linker type and content.

Film	L*	a*	b*	ΔE*	BI (%)
Control	95.422 ± 0.285^a^	−0.001 ± 0.050^a^	3.041 ± 0.181^a^	-	-
10F	92.742 ± 0.484^b^	−2.138 ±0.116^b^	20.406 ± 1.696^b^	17.705 ± 1.735^a^	22.261 ± 2.329^a^
20F	91.252 ± 0.887^c^	−1.759 ±0.513^c^	31.657 ± 3.208^c^	28.986 ± 3.208^b^	39.602 ± 5.711^b^
10AA	90.642 ± 0.492^c^	−1.510 ± 0.174^c^	31.507 ± 1.719^c^	28.910 ± 3.253^b^	39.788 ± 3.188^b^
20AA	88.835 ± 0.653^d^	−0.800 ± 0.327^d^	39.546 ± 2.248^d^	37.105 ± 2.315^c^	55.547 ± 5.008^c^

^a–d^ Two means followed by the same letter in the same column are not significantly (*P* > 0.05) different through the Tukey’s multiple range test.

**Table 4:** Tensile strength (TS) and elongation at break (EB) of gelatin films as a function of cross-linker type and content.

Film	TS (MPa)	EB (%)
Control	71.78 ± 3.82^a^	5.14 ± 0.17^a^
10F	78.62 ± 4.74^b^	5.24 ± 0.89^a^
20F	59.74 ± 2.88^c^	10.23 ± 0.46^c^
10AA	77.19 ± 4.10^a,b^	7.93 ± 0.54^b^
20AA	54.25 ± 3.28^c^	19.18 ± 0.88^d^

^a–d^ Two means followed by the same letter in the same column are not significantly (*P* > 0.05) different through the Tukey’s multiple range test.
